# Risk factors of impaired employability after cerebral venous thrombosis

**DOI:** 10.1111/cns.14083

**Published:** 2023-01-04

**Authors:** Lu Liu, Huimin Jiang, Huimin Wei, Yifan Zhou, Yan Wu, Kaiyuan Zhang, Jiangang Duan, Ran Meng, Chen Zhou, Xunming Ji

**Affiliations:** ^1^ Department of Neurology, Xuanwu Hospital Capital Medical University Beijing China; ^2^ Neurology and Intracranial Hypertension & Cerebral Venous Disease Center, National Health Commission of China, Xuanwu Hospital Capital Medical University Beijing China; ^3^ Laboratory of Brain Disorders, Ministry of Science and Technology, Collaborative Innovation Center for Brain Disorders, Beijing Capital Medical University Beijing China; ^4^ Beijing Advanced Innovation Center for Big Data‐Based Precision Medicine, School of Biological Science and Medical Engineering Beihang University Beijing China; ^5^ Beijing Institute of Geriatrics, Xuanwu Hospital Capital Medical University Beijing China; ^6^ Department of Radiology & Nuclear Medicine, Xuanwu Hospital Capital Medical University Beijing China; ^7^ Department of Emergency, Xuanwu Hospital Capital Medical University Beijing China; ^8^ Department of Neurosurgery, Xuanwu Hospital Capital Medical University Beijing China

**Keywords:** cerebral venous thrombosis, CVT recurrence, employment, outcome, sinus thrombosis

## Abstract

**Aims:**

Cerebral venous thrombosis (CVT) is a major cause of stroke in young and middle‐aged adults. This study aimed to evaluate the prevalence of post‐CVT employability decline and identify factors associated with unemployment.

**Methods:**

We identified patients first diagnosed with acute/subacute CVT at Xuanwu Hospital, Capital Medical University (January 2018 to June 2021) and invited all survivors to a clinical 6‐months follow‐up visit after onset. Baseline data were collected from all patients at admission. A modified Rankin Scale (mRS) and employment status were used to assess functional outcomes. Multivariate logistic regression was used to identify independent factors associated with unemployment.

**Results:**

A total of 303 CVT patients were eligible for this study, 131 (42.23%) patients could not return to work 6‐month after discharge. After adjusting for age and sex in multivariate analysis, motor deficits, aphasia, mental disorders, CVT recurrence, National Institutes of Health Stroke Scale (NIHSS) score at admission, and mRS 0–2 at 6‐month follow‐up were independently associated with employment after CVT. Among 263 patients whose mRS showed a favorable outcome, 102 patients were unable to return to their previous work and the risk factors for impaired ability to return to work were aphasia and CVT recurrence.

**Conclusions:**

Impaired employability after CVT was associated with motor deficits, aphasia, mental status disorders, and NIHSS score at admission. Even if they recover from CVT without physical disability, patients with a good functional prognosis have a higher risk of employment failure due to their higher rates of aphasia and CVT recurrence.

## Graphical abstract

Cerebral venous thrombosis (CVT) is a major cause of stroke in young and middle‐aged adults, and the risk factors that affect workability after CVT remain unclear. This study found that even if patients recover from CVT without physical disability, they can have a higher risk of employment failure due to their higher rates of aphasia and CVT recurrence.

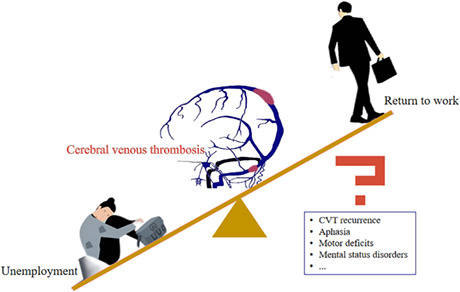

## 1 INTRODUCTION

Cerebral venous thrombosis (CVT) is an uncommon but unique form of stroke with occlusion of one or more cerebral veins or dural venous sinuses and is characterized by cerebral venous reflux disorder and intracranial hypertension. Its clinical manifestations include headache, blurred vision, epilepsy, and focal neurological dysfunction and can lead to hemorrhage or death.^1^, ^2^, ^3^ The reported incidence estimates range from approximately 13.2 per 100 million per year to 15.7 per million per year.^4^, ^5^

In the past few years, we have seen major advancements in our knowledge of the epidemiology, diagnosis, and treatment of CVT. In contrast to arterial stroke, CVT primarily affects working‐age adults, and the long‐term outcomes of most patients are favorable.^6^ Although approximately three‐quarters of patients achieve short‐term independent living, many experience residual chronic symptoms, such as persistent headache, cognitive impairment, depression, or fatigue,^7^ and the most important symptom is impaired work capacity. Previous studies evaluated that 80% of patients recover from CVT without physical disability (mRS ≤2), and 20%–40% of patients cannot return to their previous work life.^8^, ^9^, ^10^, ^11^ Currently, there are few studies on vascular events and functional outcomes after CVT, and the risk factors that affect workability after CVT remain unclear.

This study aimed to investigate the relationship between the ability to continue working after CVT and demographic characteristics, clinical manifestations, and imaging data during follow‐up in a large single‐center cohort of patients with CVT and to identify risk factors that affect employability after CVT during follow‐up.

## 2 METHODS

### 2.1 Patient identification and selection

In this retrospective cohort study, adult patients with CVT were identified from a prospective stroke registry at our center, which is national, universal, and subject to periodic audits. Clinical onset was classified as acute (<48 h), subacute (48 h–30 days), or chronic (>30 days) according to the time from symptom onset at first admission. Patients with first‐episode acute/subacute CVT enrolled in this registry between January 2018 and June 2021 were consecutively included in this study. Written consent was obtained from all participants, and the study was approved by the Ethics Committee of Xuanwu Hospital, Capital Medical University.

The inclusion criteria were as follows: acute/subacute CVT diagnosed by magnetic resonance imaging (MRI) + magnetic resonance venography (MRV), computerized tomography (CT) + CT venography (CTV), or digital subtraction angiography (DSA), and age ≥14 years. There were no restrictions on sex and patients with malignancies were excluded because they could be the direct cause of death or dependence.

### 2.2 Data collection

Baseline data were collected at admission for all patients, including etiology and risk factors for CVT, such as sex‐specific factors (use of estrogen‐progesterone, puerperium, oral contraceptives, and pregnancy), hereditary thrombophilia factors (protein C, S, or antithrombin III deficiency), acquired thrombophilia factors (antiphospholipid syndrome, nephrotic syndrome, and hyperhomocysteinemia), other risk factors (anemia, infections), and clinical symptoms and signs (headache, cerebrospinal fluid pressure, seizure, motor or sensory deficit, aphasia, metal status disorders [cognitive disturbances including abnormal alertness and orientation], and Glasgow Coma Scale [GCS] score).

The location of the thrombus (superior sagittal sinus, lateral sinus [transverse and/or sigmoid sinus], straight sinus, deep venous system, and cortical vein) and parenchymal changes (edema, infarction, and hemorrhage) were evaluated using magnetic resonance imaging combined with CT scans.

The National Institutes of Health Stroke Score (NIHSS) at admission, Modified Rankin Scale (mRS), head CTV/MRV/MRI/magnetic resonance black blood thrombus imaging video at follow‐up 6 months after discharge, regardless of whether venous sinus thrombosis recurrence occurred during follow‐up were recorded.

### 2.3 Follow‐up and clinical outcome

The regular follow‐up period was 6 months after discharge. Follow‐up and outcome data were collected using a standardized questionnaire during clinical outpatient visits. Sinus recanalization status was assessed by an experienced neuroradiologist blinded to the clinical follow‐up data, data were classified as non‐, partial, or complete recanalization according to the proposed criteria (excluding patients with cortical venous thrombosis or incomplete imaging data).^12^ The mRS score at follow‐up 6 months after discharge was used as the primary endpoint of efficacy, with an mRS score >2 indicating a poor prognosis and an mRS score ≤2 indicating a favorable outcome. CVT recurrence was recorded, which was defined as new CVT diagnosis confirmed with imaging during the six‐month follow‐up in a patient with prior CVT.^13^ The working status was evaluated and categorized as employed or unemployed. We categorized those working full time and students as employed, and those who were unable to take up any work or stop studying as unemployed. We excluded persons who retired for reasons not related to CVT (old age, other underlying illnesses, or women on maternity leave).

### 2.4 Statistics

The values of the measured parameters were checked for conformity to a normal distribution using the Kolmogorov–Smirnov test before statistical analysis. Continuous variables were expressed as mean ± SD or median with interquartile range (IQR), and categorical variables were expressed as percentages. Bivariate analysis with the t‐test or Mann–Whitney *U* test for continuous variables and the chi‐square test for categorical variables was used to identify potential variables associated with work status. Univariate analysis as independent variables analysis was performed to identify independent risk factors. Variables with a *p* < 0.05 in multivariate logistic regression, together with age and sex as covariates and unemployment as dependent variable, were entered into the regression model. We calculated odds ratios (ORs) and 95% confidence intervals (CIs) for the retained variables. A two‐sided *p*‐value <0.05 was considered significant. SPSS 22.0 for Windows (IBM Corp.) was used to analyze all data.

## 3 RESULTS

### 3.1 Baseline characteristics and outcomes in all patients with CVT

**FIGURE 1:**
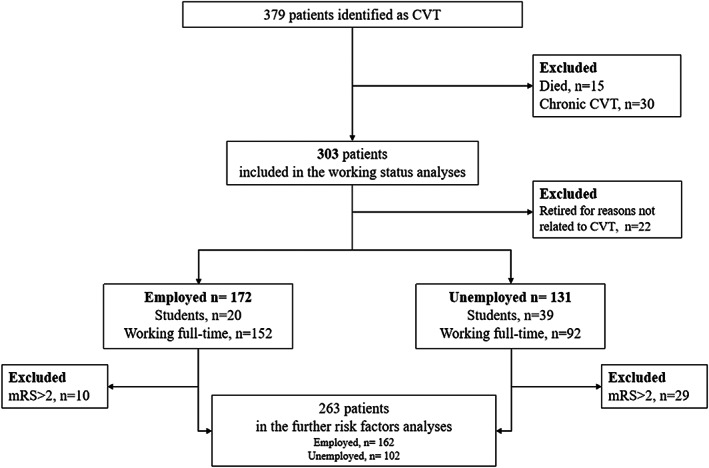
Flowchart.

We identified 379 adult patients with verified CVT diagnosis. Of these, 15 patients died and 22 patients retired for reasons not related to CVT, 30 patients were classified as chronic CVT and had accepted various treatments elsewhere, they were excluded in this study (Figure 1). As the baseline data are shown in Table 1, 303 patients with CVT were eligible for this study. The median age at the time of CVT onset was 33.66 ± 13.37 years. Most of the patients were female, accounting for 61.04%. Acquired, sex‐specific, and hereditary are the top three risk factors among these patients, and they were more likely to present symptoms including headache (94.39%), intracranial hypertension (85.15%), and seizures (37.29%). Eighty‐two patients had coma at admission and the average NIHSS score for all patients was 4.57 ± 8.312. Venous infarction and edema were present in 62.05% and 67.33% of the patients, respectively. The most common site of thrombosis in these patients is the superior sagittal sinus.

**TABLE 1:** Baseline characteristics and outcomes in all patients with CVT

Variables	All cases (*n* = 303)	Unemployment (*n* = 131)	Employment (*n* = 172)	*p*‐Value
Sex (female)	186 (61.04%)	80 (61.07%)	106 (61.63%)	0.921
Age (IQR), year	32.00 (23.00, 43.00)	34.00 (24.00, 43.00)	31.00 (22.00, 43.75)	0.555
Risk factors				
Sex‐specific	106 (34.99%)	49 (37.40%)	57 (33.14%)	0.441
Hereditary	90 (29.70%)	43 (32.82%)	47 (27.32%)	0.300
Acquired	114 (37.62%)	48 (36.64%)	66 (38.37%)	0.758
Anemia	74 (24.42%)	42 (32.06%)	32 (18.60%)	0.008*
Infection (CNS)	21 (6.90%)	8 (6.10%)	13 (7.56%)	0.606
Symptoms and signs				
Intracranial hypertension^a^	258 (85.15%)	112 (85.50%)	146 (84.88%)	0.267
Headache	286 (94.39%)	124 (94.66%)	162 (94.19%)	0.860
Seizure	113 (37.29%)	50 (38.17%)	63 (36.63%)	0.784
Motor deficits	112 (36.96%)	64 (48.85%)	48 (27.91%)	0.000^a^
Aphasia	59 (19.47%)	41 (31.30%)	18 (10.47%)	0.000*
Mental status disorders	71 (23.43%)	50 (38.17%)	21 (12.21%)	0.000*
Coma (GCS <12)	82 (27.06%)	47 (35.88%)	35 (20.35%)	0.001*
NIHSS	4.57 ± 8.312	5.96 ± 9.14	3.52 ± 7.48	0.001*
Neuroimaging at admission				
Edema	204 (67.33%)	95 (72.52%)	109 (63.37%)	0.093
Venous infarction	188 (62.05%)	91 (69.46%)	97 (56.40%)	0.020*
Hemorrhage lesion	110 (36.30%)	57 (43.51%)	53 (30.81%)	0.023*
Superior sagittal sinus	192 (63.37%)	78 (59.5%)	114 (66.28%)	0.229
Lateral sinus, right	142 (46.86%)	60 (45.80%)	82 (47.67%)	0.747
Lateral sinus left	136 (44.88%)	57 (43.51%)	79 (45.93%)	0.675
Deep cerebral venous	75 (24.75%)	35 (26.72%)	40 (23.26%)	0.490
Cortex venous	170 (56.10%)	81 (61.83%)	89 (51.74%)	0.080
Outcome				
mRS, 0**–**2	263 (86.80%)	102 (77.86%)	161 (93.60%)	0.000*
Non‐Recanalization^b^	65 (22.89%)	26 (20.63%)	39 (24.68%)	0.043*
CVT recurrence	30 (9.90%)	19 (14.50%)	11 (6.40%)	0.017*

Abbreviations: CNS, central nervous system; CVT, cerebral venous thrombosis; GCS, Glasgow Coma Scale; IQR, interquartile range; mRS, Modified Rankin Scale; NIHSS, National Institutes of Health Stroke Scale.

^a^ Intracranial hypertension was identified according to the result of the first lumbar puncture after hospitalization (>180 mm H_2_0).

^b^ A total of 284 patients were evaluated after excluding 19 patients with cortical venous thrombosis (14 in the employment group and five in the unemployment group).

Asterisks mean a significant statistical difference according the *p*‐value < 0.05.

Of the survivors who had completed the 6‐month follow‐up, 86.80% of them had good outcomes with an mRS 0–2. Sixty‐five patients (22.89%) showed nonrecanalization according to follow‐up images, and 30 (9.90%) experienced CVT recurrence during follow‐up.

In the univariate analysis, we compared baseline factors between the unemployment and employment groups. Anemia seemed to be related to working status (*p* = 0.008), and unemployed patients had a greater likelihood of presenting symptoms that included motor deficits, aphasia, mental status disorders, coma (GCS <12), and a higher NIHSS score. The imaging characteristics showed that unemployed patients had a higher proportion of venous infarction (69.46%) and hemorrhage (43.51%) at admission. At 6 months of follow‐up, 263 (86.80%) survivors achieved favorable functional outcomes (mRS, 0–2), and the employment group had a higher proportion of favorable functional outcomes (161 [93.60%] vs. 102 [77.86%], *p* = 0.000). A total of 65 (22.89%) survivors exhibited venous sinus vessel nonrecanalization. The employment group appeared to have a higher likelihood of nonrecanalization (39 [24.68%] vs. 26 [20.63%], *p* = 0.043), while the unemployment group had a significantly higher percentage of CVT recurrence (19 [14.50%] vs. 11 [6.40%], *p* = 0.017).

### 3.2 Risk factors for unemployment in all CVT survivors

Multivariate logistic regression analysis was performed to identify independent risk factors for unemployment among all CVT survivors. In addition to age and sex, risk factors that showed significant differences, including motor deficits, aphasia, mental status disorders, NIHSS score, coma (GCS <12), CVT recurrence, recanalization status, and mRS score (0–2) as the dependent variables were entered into the regression model.

In the multivariate logistic regression model, motor deficits (OR, 2.444; 95% CI, 1.210–4.936; *p* = 0.013), aphasia (OR, 3.634; 95% CI, 1.507–8.765, *p* = 0.004), mental disorders at diagnosis (OR, 3.117; 95% CI, 1.392–6.982, *p* = 0.006), NIHSS score at diagnosis (OR, 0.928; 95% CI, 0.874–0.986; *p* = 0.016), CVT recurrence (OR, 4.770; 95% CI, 1.846–12.326; *p* = 0.001), and mRS 0–2 (OR, 0.144; 95% CI, 0.029–0.713, *p* = 0.018) were independently associated with unemployment (Table 2).

**TABLE 2:** Risk factors for unemployment CVT survivors

	OR	95% CI	*p*
Female	0.686	0.390–1.206	0.190
Age	1.003	0.983–1.024	0.753
Anemia	1.028	0.695–1.522	0.888
Motor deficits	2.444	1.210–4.936	0.013*
Aphasia	3.634	1.507–8.765	0.004*
Mental status disorders	3.117	1.392–6.982	0.006*
NIHSS	0.928	0.874–0.986	0.016*
Coma (GCS <12)	1.075	0.457–2.530	0.868
Venous infarction	0.725	0.353–1.490	0.382
Hemorrhage lesion	1.236	0.603–2.534	0.563
CVT recurrence	4.770	1.846–12.326	0.001*
No recanalization	1.333	0.961–1.847	0.085
mRS (0–2)	0.144	0.029–0.713	0.018*

Abbreviations: CIs, confidence intervals; CVT, cerebral venous thrombosis; GCS, Glasgow Coma Scale; mRS, Modified Rankin Scale; NIHSS, National Institutes of Health Stroke Scale; ORs, odds ratios.

Asterisks mean a significant statistical difference according the *p*‐value < 0.05.

### 3.3 Baseline characteristics in patients with mRS 0–2

We analyzed the baseline factors associated with unemployment in patients with mRS 0–2 (*n* = 263; 161 in the employment group and 102 in the unemployment group). The employed group showed fewer serious functional impairments, including motor deficits, aphasia, and mental status disorders (*p* < 0.05). However, the unemployed group had a significantly higher proportion of motor deficits (41 [40.20%] vs. 43 [26.71%], *p* = 0.023), a higher likelihood of aphasia (25 [24.51%] vs. 15 [9.32%], *p* = 0.001), and mental status disorders (29 [28.43%] vs. 18 [11.18%], *p* = 0.000); Table 3.

**TABLE 3:** Baseline characteristics and outcomes of employment and unemployment in patients with mRS 0–2

Variables	All cases (*n* = 263)	Unemployment (*n* = 102)	Employment (*n* = 161)	*p*‐Value
Sex (female)	156 (59.32%)	56 (54.90%)	100 (62.11%)	0.247
Age (IQR), year	31.00 (22.00,45.00)	28.00 (22.00,42.00)	32.00 (22.00,44.00)	0.688
Risk factors				
Sex specific	87 (33.08%)	34 (33.33%)	53 (32.92%)	0.945
Hereditary	78 (29.66%)	34 (33.33%)	44 (27.33%)	0.300
Acquired	98 (37.26%)	40 (39.22%)	58 (36.02%)	0.603
Anemia	57 (21.67%)	27 (26.47%)	30 (18.63%)	0.134
Infection (CNS)	16 (6.08%)	4 (3.92%)	12 (7.45%)	0.240
Symptoms and signs				
Intracranial hypertension^a^	198 (83.89%)	76 (81.72%)	122 (85.31%)	0.567
Headache	251 (95.44%)	95 (93.14%)	156 (96.89%)	0.232
Seizure	92 (34.98%)	37 (36.27%)	55 (34.16%)	0.727
Motor deficits	84 (31.94%)	41 (40.20%)	43 (26.71%)	0.023*
Aphasia	40 (15.21%)	25 (24.51%)	15 (9.32%)	0.001*
Mental status disorders	47 (17.87%)	29 (28.43%)	18 (11.18%)	0.000*
Coma (GCS <12)	49 (18.63%)	23 (22.55%)	26 (16.15%)	0.154
NIHSS	3.47 ± 6.502	2.86 ± 6.02	3.80 ± 6.79	0.066
Neuroimaging at admission				
Edema	168 (63.87%)	70 (68.63%)	98 (60.87%)	0.203
Venous infarction	152 (57.79%)	66 (64.71%)	86 (53.42%)	0.071
Hemorrhage lesion	74 (28.14%)	32 (31.37%)	42 (26.09%)	0.354
Superior sagittal sinus	167 (63.50%)	61 (59.80%)	106 (65.84%)	0.323
Lateral sinus, right	127 (48.29%)	50 (49.02%)	77 (47.83%)	0.851
Lateral sinus left	112 (42.59%)	40 (39.22%)	72 (44.72%)	0.380
Deep cerebral venous	60 (22.81%)	23 (22.55%)	37 (22.98%)	0.935
Cortex venous	144 (54.75%)	61 (59.80%)	83 (51.55%)	0.191
Outcomes				
Non‐recanalization^b^	60 (24.59%)	23 (23.71%)	37 (25.17%)	0.211
CVT recurrence	28 (10.65%)	18 (17.65%)	10 (6.21%)	0.003*

Abbreviations: CNS, central nervous system; CVT, cerebral venous thrombosis; GCS, glasgow coma scale; IQR, interquartile range; mRS, Modified Rankin Scale; NIHSS, National Institutes of Health Stroke Scale.

^a^ A total of 236 patients with intracranial hypertension were included (143 in the employment group and 93 in the unemployment group).

^b^ A total of 244 patients were evaluated after excluding 19 patients with cortical venous thrombosis (14 in the employment group and five in the unemployment group).

Asterisks mean a significant statistical difference according the *p*‐value < 0.05.

### 3.4 Risk factors for unemployment in CVT survivors with mRS 0–2

A multivariate logistic regression analysis was performed to identify independent risk factors for unemployment in CVT survivors with mRS 0–2. We also entered risk factors that showed significant differences, as well as those considered relevant in Table 3 after adjusting for age and sex.

Among the independent variables included in the model, aphasia (OR, 2.586; 95% CI, 1.153–5.800, *p* = 0.021) and CVT recurrence (OR, 3.711; 95% CI: 1.569–8.777; *p* = 0.003) were independently associated with unemployment status in CVT survivors with favorable outcomes at the 6 months follow‐up (mRS 0–2) (Table 4).

**TABLE 4:** Risk factors of unemployment in CVT survivors with mRS 0–2

Potential risk factors	OR	95% CI	*p*
Female	0.620	0.356–1.082	0.092
Age	0.992	0.972–1.012	0.441
Motor deficits	1.569	0.862–2.856	0.141
Aphasia	2.586	1.153–5.800	0.021*
Mental status disorders	1.985	0.980–4.021	0.057
CVT recurrence	3.711	1.569–8.777	0.003*

Abbreviations: CIs, confidence intervals; CVT, cerebral venous thrombosis; mRS, modified Rankin Scale; ORs, odds ratios.

Asterisks mean a significant statistical difference according the *p*‐value < 0.05.

## 4 DISCUSSION

This study highlights that patients with a good functional prognosis, even if they recover from CVT without physical disability, have a higher risk of employment failure due to their higher rates of aphasia and CVT recurrence. Our study provides data on various aspects of admission and short‐term follow‐up in a single‐center cohort of 303 patients with CVT and found that motor deficits, aphasia, mental status disorders, NIHSS score, CVT recurrence, and mRS >2 were associated with impaired work ability. In particular, for patients who achieved functional independence (mRS 0–2), aphasia (OR, 2.586; 95% CI, 1.153–5.800; *p* = 0.021), and CVT recurrence (OR, 3.711; 95% CI, 1.569–8.777; *p* = 0.003) were independently associated with unemployment status at 6 months of follow‐up.

Only a few previous studies have explored the presence of work‐related problems after CVT, focusing more on its relationship with cognitive impairment. Bruijn et al. conducted a mean follow‐up of 18.5‐month and examined the cognition and functional health of 47 patients with CVST. In a total of 19 (40%) survivors who did not resume previous work, 17 (89%) of them had a mild or moderate handicap (mRS ≤2) with almost half of the cognitive impairments.^14^ A later two‐center observational study showed similar results and found that the only factor associated with work failure was defective cognitive status (OR, 21.0; 95% CI, 3.35–131.44; *p* = 0.001).^10^ However, in another study of 34 patients with CVST, after long‐term follow‐up (median, 3.5 years), all patients recovered without functional disability, and all 34 patients could resume their previous level of economic activity regardless of the mild neurological signs and cognitive impairment some of them showed.^11^ None of the above studies directly assessed long‐term working‐related outcomes after CVT, and all studies assessed employment status at cross‐sectional follow‐up.^10^, ^11^, ^14^

Before our study, few studies emphasized vocational outcomes after CVT during the follow‐up period.^9^, ^15^ One was conducted by Hiltunen et al., in which 91 (75.2%) of 121 patients who had vocational outcome results were employed at follow‐up (median: 39 months).^9^ In another smaller study, 44 (71.0%) patients returned to work during follow‐up (34 patients in the first year) and 18 (29.0%) never regained their ability to work.^15^

We found several possible risk factors for unemployment after CVT, such as NIHSS score, mRS score >2, and dysfunction, including motor dysfunction and aphasia at admission. Hiltunen et al. observed that an NIHSS score greater than 2 points was strongly associated with incomplete functional recovery and diminished working ability. They suggested that the NIHSS score be used for early screening of the risk of poor prognosis in CVT.^9^ However, in our study, higher NIHSS score cannot directly related to the unfavorable working status after CVT. We think this may be explained as NIHSS score can be more useful when patients had in‐hospital symptoms of neurological deficits such as aphasia or motor deficits. Many CVT patients with other typical symptoms in our center, for instance, severe headache and intracranial hypertension (>330 mm H_2_O), had not achieved high NIHSS score at admission while their working ability were actually affected to varying degrees. An mRS score >2 is commonly used to define bad outcomes in CVT studies.^8^ Therefore, at current stage, NIHSS and mRS scores should be used together when indicating rehabilitation difficulties and ability to returning to work of CVT patients. In the future, a better objective assessment may be available when assessing the severity and prognosis of CVT.

In particular, we explored the relationship between CVT recurrence and continued employment. CVT recurrence may interrupt patients' normal work and reduce their motivation to work. In contrast, the recurrence of CVT in our study was higher than that observed in previous studies, in which 2.2%–4.4% of patients suffered from CVT,^16^, ^17^ the different incidence of recurrent CVT in our study may be due to the fact that our center, as the China National Clinical Research Center for Neurological Diseases and the National Cerebral Venous Disease Centers of China, admits a higher proportion of patients with refractory or severe CVT.

An mRS score ≥2, reflecting a state of functional independence, is generally considered a good outcome.^18^ In a small‐sample study conducted by Koopman et al., of the 38 previously employed patients who had an mRS score ≤2, eight (21%) did not return to work, and 13 (34%) returned and found their jobs more difficult.^8^ Our study strengthens the notion that the use of mRS to assess outcomes in CVT may not adequately emphasize the importance of some common but less obvious effects of CVT, such as reduced work ability.

Still, socioeconomic factors such as retirement and unemployment benefit, insurance policy or sexual bias can affect the people's willingness to work. National and regional differences may also contribute to different outcomes. Studies including larger cohorts from countries with markable different sickness pension and social welfare are necessary in the future.

Our study has some limitations. First, we did not collect residual symptoms from patients during follow‐up and other possible factors, such as education level and anticoagulant use. Therefore, these aspects could not be evaluated and compared with those of existing studies, which caused some omissions in our study. Second, since this was a retrospective study, there was a risk of loss of patients and selection bias. Our clinical follow‐up is only 6 months. Therefore, more convincing evidence of long‐term follow‐up cannot be obtained. Third, during our follow‐up, all the patients in the employment group were able to continue their previous work without substitution for less intensive work, whether there is a difference in the working ability of these patients needs to be further evaluated. Longer follow‐up period may demonstrate the long‐term negative impact of CVT on work capacity. Finally, the imaging parameters used during follow‐up in our study were relatively simple, including two common parameters, the non‐recanalization rate and recurrence. Many advanced imaging techniques and methods have been applicated in the cerebral venous system diseases, which helped to discover the important role of venules in brain parenchymal lesions, cerebral small vessel disease (cSVD) and even age‐related neurodegenerative processes.^19^, ^20^, ^21^, ^22^ However, it has not been reported whether the intracranial hypertension caused by the non‐recanalization of the venous sinus after CVT affects the venules and deep medullary veins. In the future study, potential imaging indicators such as drainage of small venules and deep medullary veins can be included for more accurate prognosis after CVT. It is also worth further exploring whether the location and quantity of vascular recanalization are related to the prognosis of working ability. The main advantage of our study was that we had a CVT cohort of 303 patients with relatively complete clinical information, which allowed us to analyze the prognosis of this rare disease. Despite its retrospective limitations, the present study provides valuable empirical evidence to assess the work capacity of patients with CVT.

## 5 CONCLUSIONS

Our study reinforces the finding that impaired employability after CVT was associated with motor deficits, aphasia, mental status disorders, and NIHSS score at admission. Even if they recover from CVT without physical disability, patients with a good functional prognosis have a higher risk of employment failure due to their higher rates of aphasia and CVT recurrence.

## AUTHOR CONTRIBUTIONS

XMJ and CZ: conceptualization (lead), review, and editing (lead). LL: writing—original draft (lead), formal analysis (lead), writing—review and editing (equal). HMJ, HMW, and YFZ: data curation (lead), formal analysis (supporting), writing—review and editing (supporting). KYZ, JGD, and RM: writing—review and editing (supporting). YW and XMJ: funding acquisition (lead). All authors contributed to the article and approved the submitted version.

## FUNDING INFORMATION

This study was supported by the Cheung Kong (Changjiang) Scholars Program (T2014251) and the Pharmaceutical Collaboration Project of the Beijing Science and Technology Commission (Z181100001918026).

## CONFLICT OF INTEREST

The authors declare that they have no conflicts of interest.

## Data Availability

Data available on request from the authors.
